# Lack of the Long Pentraxin PTX3 Promotes Autoimmune Lung Disease but not Glomerulonephritis in Murine Systemic Lupus Erythematosus

**DOI:** 10.1371/journal.pone.0020118

**Published:** 2011-05-27

**Authors:** Maciej Lech, Christoph Römmele, Onkar P. Kulkarni, Heni Eka Susanti, Adriana Migliorini, Cecilia Garlanda, Alberto Mantovani, Hans-Joachim Anders

**Affiliations:** 1 Medizinische Poliklinik, University of Munich, Munich, Germany; 2 Istituto Clinico Humanitas, IRCCS, Rozzano, Italy; 3 Department of Translational Medicine, University of Milan, Rozzano, Italy; University of California, San Francisco, United States of America

## Abstract

The long pentraxin PTX3 has multiple roles in innate immunity. For example, PTX3 regulates C1q binding to pathogens and dead cells and regulates their uptake by phagocytes. It also inhibits P-selectin-mediated recruitment of leukocytes. Both of these mechanisms are known to be involved in autoimmunity and autoimmune tissue injury, e.g. in systemic lupus erythematosus, but a contribution of PTX3 is hypothetical. To evaluate a potential immunoregulatory role of PTX3 in autoimmunity we crossed *Ptx3*-deficient mice with *Fas*-deficient (lpr) C57BL/6 (B6) mice with mild lupus-like autoimmunity. PTX3 was found to be increasingly expressed in kidneys and lungs of B6*lpr* along disease progression. Lack of PTX3 impaired the phagocytic uptake of apoptotic T cells into peritoneal macrophages and selectively expanded CD4/CD8 double negative T cells while other immune cell subsets and lupus autoantibody production remained unaffected. Lack of PTX3 also aggravated autoimmune lung disease, i.e. peribronchial and perivascular CD3+ T cell and macrophage infiltrates of B6*lpr* mice. In contrast, histomorphological and functional parameters of lupus nephritis remained unaffected by the *Ptx3* genotype. Together, PTX3 specifically suppresses autoimmune lung disease that is associated with systemic lupus erythematosus. Vice versa, loss-of-function mutations in the *Ptx3* gene might represent a genetic risk factor for pulmonary (but not renal) manifestations of systemic lupus or other autoimmune diseases.

## Introduction

Systemic lupus erythematosus (SLE) involves polyclonal autoimmunity against multiple nuclear autoantigens and presents clinically in a broad spectrum of manifestations ranging from mild fever, skin rashes, and arthralgia to severe inflammation of kidney, lungs, or brain [1]. It has become evident that SLE is not a single disease with a uniform trigger but rather a syndrome that can develop from many different causes [2]. The pathogenesis of SLE is largely based on variable combinations of genetic variants that promote loss-of-tolerance or tissue inflammation [2], [3]. For example, some gene affect apoptosis, opsonization of dying cells, phagocytosis or the digestion of self-DNA which increase the exposure of nuclear particles to the immune system [4]. Another set of risk genes enhance the immune recognition of self nucleic acids by Toll-like receptors (TLR) in dendritic cells which increases the production of type I interferon [5], [6] and eventually the expansion of autoreactive lymphocytes [7]. A third class of genetic lupus risk factors affects tissue inflammation [4].

Pentraxins belong to the first (and the third) group of molecules. The short pentraxins, C-reactive protein (CRP) and serum amyloid P (SAP), are acute phase proteins that are strongly induced in hepatocytes in response to IL-6 [8]. CRP and SAP bind to all types of microorganisms, dead cells, and other particles and facilitate complement–mediated killing as well as uptake of the particle into phagocytes [8]. As such the short pentraxins foster the rapid clearance of pathogens and dead cells from the extracellular space [9]. The latter is particularly important in order to prevent an exposure of nuclear particles to the immune system [10]. In analogy to complement deficiency genetic lack of CRP or SAP is associated with impaired clearance of apoptotic cells and the onset of lupus [11], [12], [13]. Pentraxin gene polymorphisms are unlikely to account broadly for human SLE but, interestingly, serum CRP and SAP levels are usually low in the majority of lupus patients in the absence of infection despite significant SLE activity [14], [15]. This has been attributed to anti-pentraxin antibodies as well as to the suppressive effect of IFN-α on the promotor activity of the short-pentraxins [16], [17]. A recent study also examined serum levels of the long pentraxin PTX3 which were high in patients with all kinds of rheumatic diseases but remain low in patients affected by SLE [18], [19]. Anti-PTX3 antibodies behaved the opposite way [18], [19].

The long pentraxin PTX3, in contrast to the short pentraxins, is produced outside the liver by neutrophils, macrophages, myeloid dendritic cells, as well as a number of non-immune cells in response to IL-1, TNF-α, and TLR agonists [20]. PTX3 shares some immunomodulatory functions with the short pentraxins such as binding to C1q and activation of the classical complement pathway [21], and inhibiting the amplification loop of the alternative complement pathway [22], and accelerating host defense to pathogens [8]. However, PTX3 seems to also have unique immunoregulatory functions such as modulating the phagocytic uptake of apoptotic cells by macrophages and dendritic cells [23], [24], [25], [26], and interacting with P-selectin which inhibits leukocyte recruitment [27]. Although all of the aforementioned mechanisms might be involved in the pathogenesis of autoimmune diseases, a contribution of PTX3 to SLE is speculative to date. PTX3 might promote SLE via modulating the clearance of apoptotic cells or by driving complement-mediated tissue pathology. PTX3 might also protect from SLE manifestations by suppressing P-selectin-mediated leukocyte recruitment to affected organs.

In order to test whether *Ptx3* serves as a modifier gene on established SLE we generated *Ptx3*-deficient C57BL/6*^lpr/lpr^* (B6*^lpr^*) mice and compared the phenotype with wild type B6*^lpr^* mice, an autoimmune mouse strain that develop lupus autoantibodies but only mild SLE manifestations in kidneys and lung [28].

## Results

### Lack of PTX3 impairs the clearance of apoptotic cells

**Figure 1:**
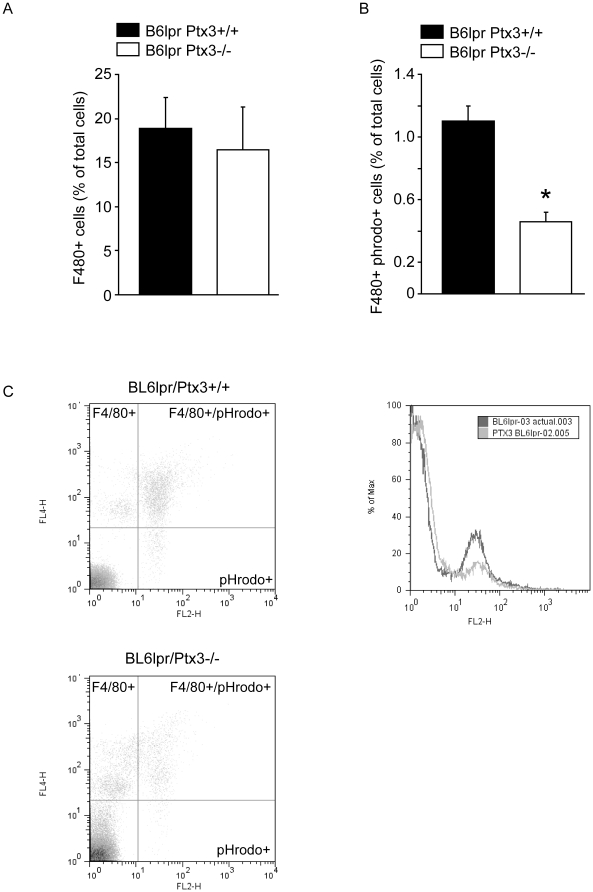
PTX3 fosters the phagocytic uptake of apoptotic cells. A: 2×10^5^ pHrodo-labeled apoptotic cells were injected intraperitoneally into B6*lprPtx3*-deficient and B6*lpr* wildtype mice and 45 min later peritoneal lavage fluids where prepared for flow cytometry. Figure A displays the percentage of F4/80+ peritoneal macrophages of all cells in lavage fluids which where not different in B6*lpr* (black bar) from B6*lpr Ptx3*-deficient mice (white bar). Figure B displays the number of F4/80+ cells also positive for pHrodo, a dye which gets only activated in the acidic environment of phagolysosomes. Data represent means ± SEM from three independent experiments; * p<0.05 versus wild type. Figure C displays representative dot plots from this experiment. C: Representative density plots of F4/80+Phrodo+ cells in B6*lprPtx3*-deficient and B6*lpr* wildtype mice.

PTX3 was reported to regulate the C1q-mediated phagocytosis of apoptotic cells *in vitro* [21]. As impaired clearance of apoptotic cells is a well established pathomechanism of SLE [29] we first tested the ability of *Ptx3*-deficient mice to clear apoptotic cells by phagocytosis *in vivo*. PHrodo-labeled apoptotic cells were injected into the peritoneal cavities of *Ptx3*-deficient and wild type B6*lpr* mice. After 45 minutes peritoneal lavage fluids were analyzed by flow cytometry for pHrodo+ F4/80 macrophages. F4/80+ peritoneal macrophages of *Ptx3*-deficient mice displayed a significantly reduced capacity to take up apoptotic cells as compared to wild type B6*lpr* mice (Figure 1). Thus, lack of PTX3 is associated with a reduced clearance of apoptotic cells.

### PTX3 expression in autoimmune B6*^lpr^* mice

**Figure 2:**
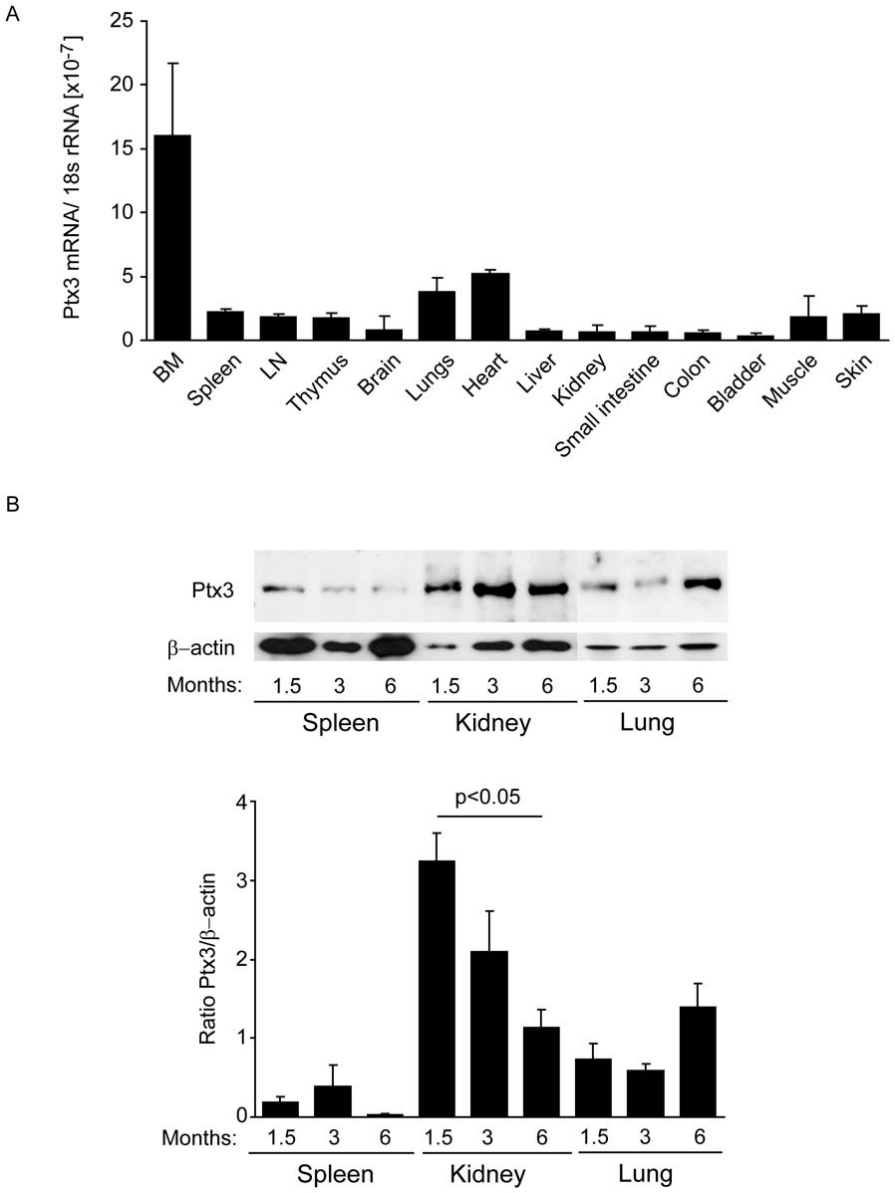
PTX3 expression in mice. A: RNA was isolated from organs of 6 week old B6*^lpr^* mice for real-time RT-PCR. Data are expressed as means of the ratio of the specific mRNA versus that of 18S rRNA ± SEM. B: Protein samples were prepared from spleens, kidneys, and lungs of B6*^lpr^* mice at 1, 3, and 6 months of age. PTX3 Western blot indicates the quantitiative (20 µg protein load per lane) PTX3 protein expression in each organ over time. The histogram represents the ratio of PTX3 expression to the expression of the β-actin loading control. Data represent means ± SEM from three independent experiments.

Next we characterized the expression of PTX3 mRNA in solid organs of 6 week old B6*lpr* mice. PTX3 mRNA was highly expressed in bone marrow (Figure 2A). Among the solid organs PTX3 mRNA levels were much higher heart and lungs as compared to the kidneys and the urinary bladder. PTX3 protein expression was confirmed by Western blot in spleens, kidneys and lungs of B6*lpr* mice. Spleen and kidney PTX3 expression decreased over time when autoimmunity progresses in B6*lpr* mice (Figure 2B). By contrast, PTX3 protein expression increased in lungs at 6 months of age in B6*lpr* mice indicating local production of PTX3 in lungs which are usually affected by autoimmune tissue injury in B6*lpr* mice. Thus, PTX3 is increasingly expressed in lungs during the progression of autoimmunity of B6*lpr* mice.

### PTX3 suppresses lymphoproliferation in B6*^lpr^* mice

**Figure 3:**
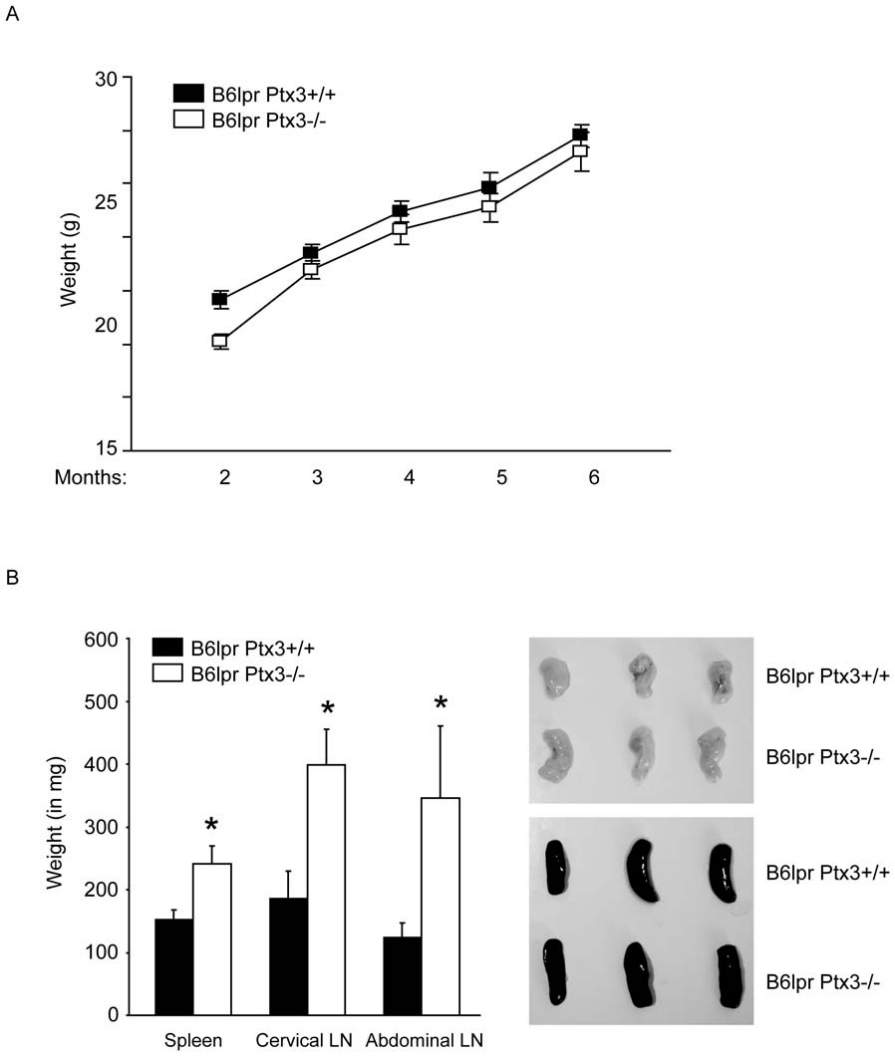
PTX3 and lymphoproliferation in B6*^lpr^* mice. A: Body weight increased similarly over time in *Ptx3*-deficient or wild type B6*^lpr^* mice. Data are means ± SEM from at least 18 mice in each group. B: At 6 months of age *Ptx3*-deficient B6*^lpr^* mice revealed splenomegaly and hyperplasia of cervical lymph nodes as compared to age-matched B6*^lpr^* control mice. Quantitative data on spleen and lymphnode weights are means ± SEM from at least 18 mice in each group, * p<0.05 versus B6*^lpr^* mice.

Next we generated *Ptx3*-deficient B6*^lpr^* mice. The autoimmune phenotype of homozygous B6*^lpr^* mice is introduced only by mutation of a single lupus susceptibility gene (*lpr*) which impairs Fas-induced apoptosis of autoreactive B and T cells [28]. Because B6*^lpr^* mice develop only mild autoimmune syndrome, litters of B6*^lpr/Ptx3−/−^* mice could be bred along Mendelian ratios from B6*^lpr/Ptx3+/−^* mice and revealed no differences in body weight gain between the two genotypes (Figure 3A). For SLE phenotype analysis we first evaluated the size of spleens and lymph nodes in 6 months old B6*^lpr^* and B6*^lpr/Ptx3−/−^* mice. Spleens and lymph nodes were significantly enlarged in B6*^lpr/Ptx3−/−^* mice as compared to B6*^lpr^* mice. This was evident from spleen and cervical lymph node weights (Figure 3B).

### PTX3 specifically suppresses CD4/CD8 double negative T cells in B6*^lpr^* mice

**Figure 4:**
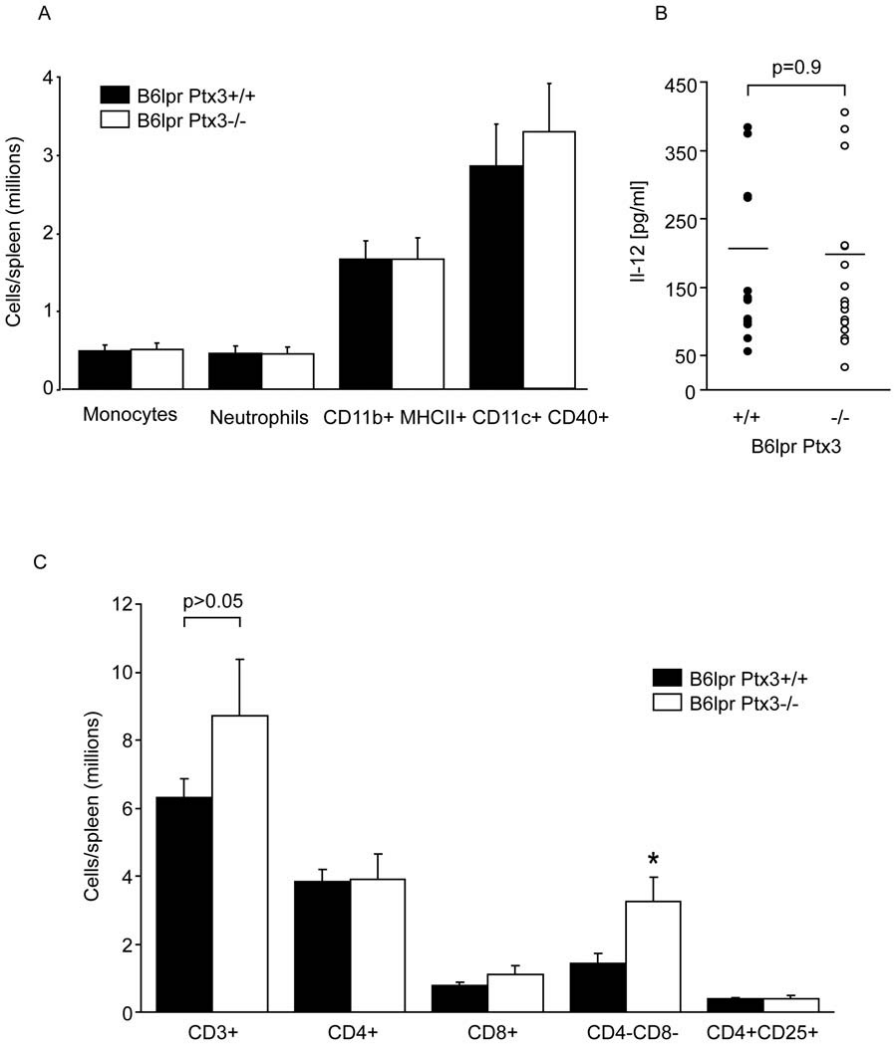
Lack of PTX3 specifically affects T cells subsets in B6*^lpr^* mice. A: Spleen monocytes, neutrophils, CD11b+ and CD11c+ cells were quantified in B6*^lpr/Ptx3−/−^* and B6*^lpr^* wild-type mice by flow cytometry also using surface MHCII and CD40 expression as a marker of cell activation as described in methods. Data represent means ± SEM from 10 mice in each group. B: Serum IL-12p40 levels were determined in 6 months old B6*^lpr^* of both genotypes by ELISA and are shown as dot blot. C: Flow cytometry was used to determine the total number of distinct T cell subsets in spleens of 6 months old B6*^lpr/Ptx3−/−^* and B6*^lpr^* wild type mice. The graphs in B–C present means ± SEM of 8–14 mice in each group * p<0.05.

Because we had found *Ptx3*-deficiency impaired the rapid clearance of apoptotic cells we hypothesized that sustained exposure to dead cells would modulate the activation of dendritic cells, cells that handle lupus autoantigens and drive the expansion of autoreactive lymphocytes in SLE, like in E8-Mag or DNAse 1-deficient mice [30], [31]. We performed flow cytometry to quantify and characterize the activation state of CD11c+ dendritic cells without additional stimuli directly after the spleen harvest at 6 months of age. However, the numbers of CD11c+/CD40+ or the CD11b/MHCII+ cells were identical as well as the total numbers of monocytes or neutrophils in both genotypes (Figure 4A). Consistent with this finding serum levels of IL-12 were unaffected by the PTX3 genotype (Figure 4B). Does lack of PTX3 affect T cell populations in B6*^lpr^* mice? The numbers of CD4/CD8 double negative ‘autoreactive’ T cells were increased in *Ptx3*-deficient B6*^lpr^* mice (Figure 4C). In contrast, the numbers of all other T cell subsets, i.e. CD4+, CD8+, and CD4+/CD25+ T cells were comparable between the two genotypes (Figure 4C). Thus, PTX3-deficiency is associated with a selective expansion of CD4/CD8 double negative T cells in B6*^lpr^* mice.

### PTX3 does not regulate B cell expansion and autoantibody production in B6*^lpr^* mice

**Figure 5:**
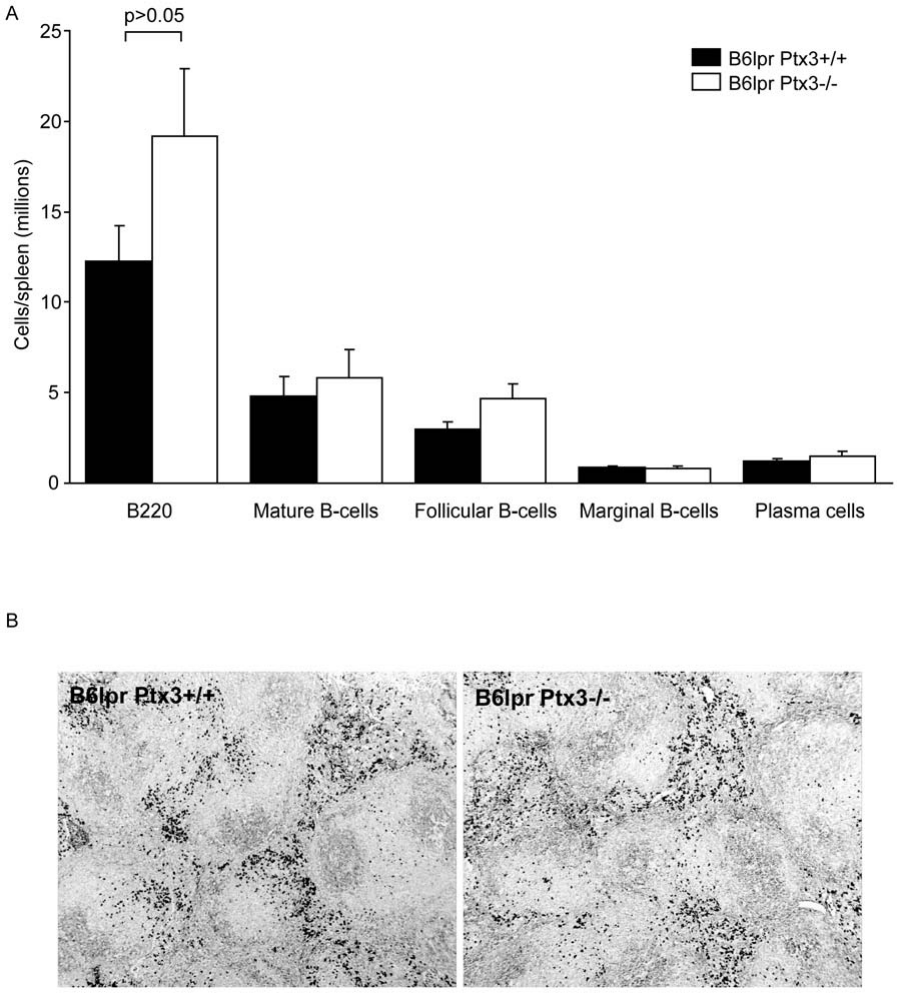
PTX3 and B cell subsets in B6*^lpr^* mice. A: Flow cytometry was used to determine the total number of distinct B cell subsets in spleens of 6 months old B6*^lpr/Ptx3−/−^* and B6*^lpr^* wild type mice. The histogram presents means ± SEM of 8–14 mice in each group. B: Spleens from the same mice were stained for IgM to localize plasma cell areas. The images are representative for 6 mice in each group. Original magnification ×100.

**Figure 6:**
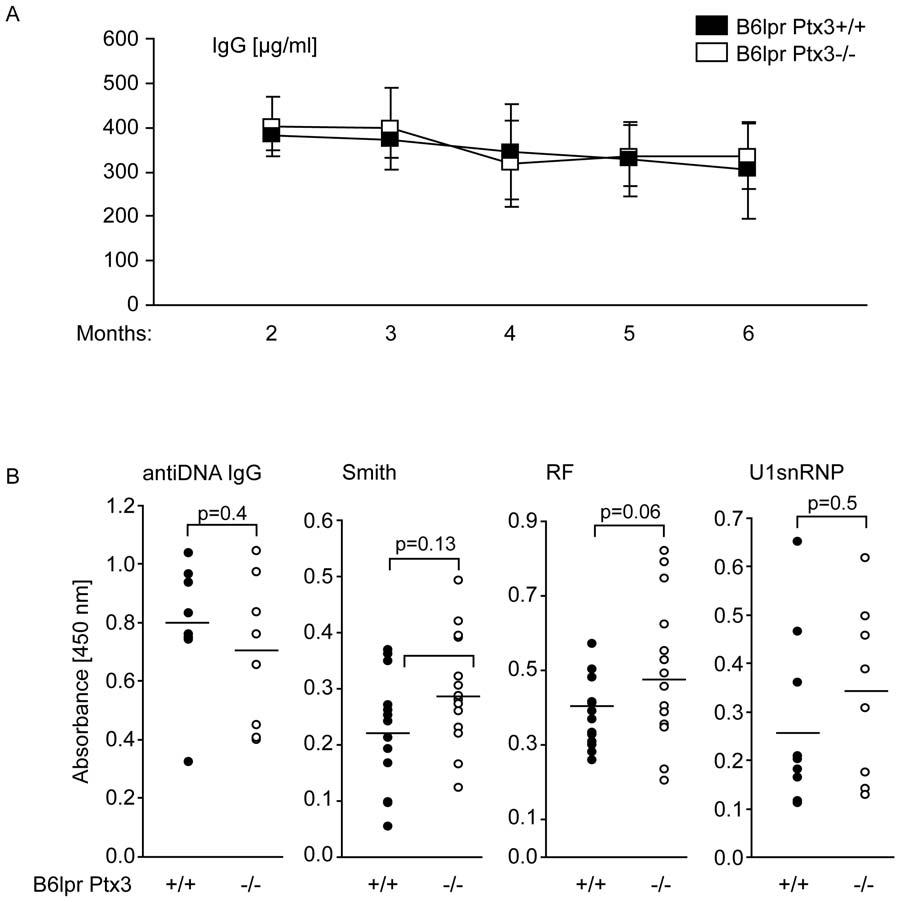
PTX3 and humoral immunity in lupus of B6*^lpr^* mice. B6*^lpr/Ptx3−/−^* (□) and B6*^lpr^* wild-type mice (▪) were bled at monthly intervals to determine serum levels of IgG (A). Data represent means ± SEM from at least 12 mice at each time point and genotype. B: DsDNA autoantibodies and various lupus autoantibodies were analysed by ELISA at 6 months only and are shown as dot blots. 6 mice at each time point and genotype. No significant differences were detected between the two genotypes for any of the parameters.

Flow cytometry did not reveal any difference in numbers of mature B cells, follicular B cells, marginal zone B cells, and plasma cells in spleens (Figure 5A) which was consistent with the comparable size of the IgM+ plasma cell areas in spleens of B6*^lpr^* and B6*^lpr/Ptx3−/−^* mice (Figure 5B). Consistent with the numbers of B cells, *Ptx3*-deficient B6*^lpr^* mice displayed similar serum IgG levels as compared to 6 months old B6*^lpr^* wild-type mice (Figure 6A). At that time lack of PTX3 did also had no effect on serum dsDNA autoantibody levels from total IgG, IgG1, IgG2a/c, and IgG3. The specificity of dsDNA autoantibodies was confirmed by using the *Critidiae luciliae* assay. Diluted serum from B6*^lpr/Ptx3−/−^* mice showed comparable binding to the dsDNA of the flagellate's kinetoplast as serum of B6*^lpr^* mice (not shown). In addition, lack of PTX3 did not affect the levels of anti-Sm IgG, anti-U1snRNP IgG, and rheumatoid factor as compared to B6*^lpr^* mice (Figure 6B). In addition, *Ptx3*-deficient B6 mice did not reveal any sign of spontaneous autoimmunity, e.g. autoantibodies against ANA, dsDNA or rheumatoid factor up to 12 months of age. Thus, PTX3 is redundant for the expansion of B cells and plasma cells as well as for the production of numerous autoantibodies against nuclear autoantigens in B6*^lpr^* mice lack of PTX3 alone does not induce autoimmunity against DNA.

### PTX3 suppresses autoimmune lung but not kidney disease in B6*^lpr^* mice

**Figure 7:**
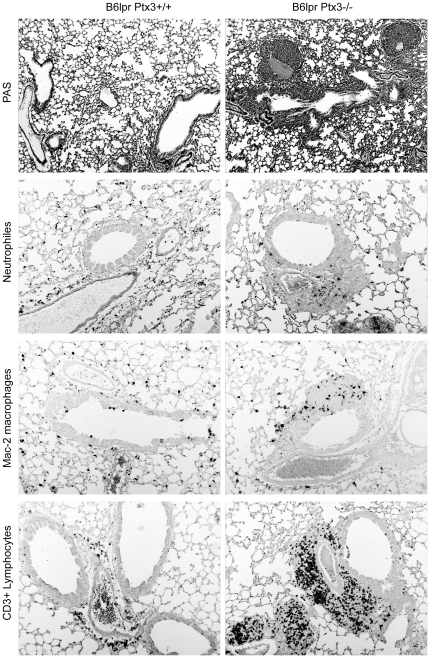
PTX3 and lung injury in B6*^lpr^* mice. Lung sections were stained either with PAS or with specific antibodies for neutrophils, macrophages or T lymphocytes. Images are representative for at least 12 mice in each group. Original magnification ×100 (PAS) and ×200 (immunostaining).

**Figure 8:**
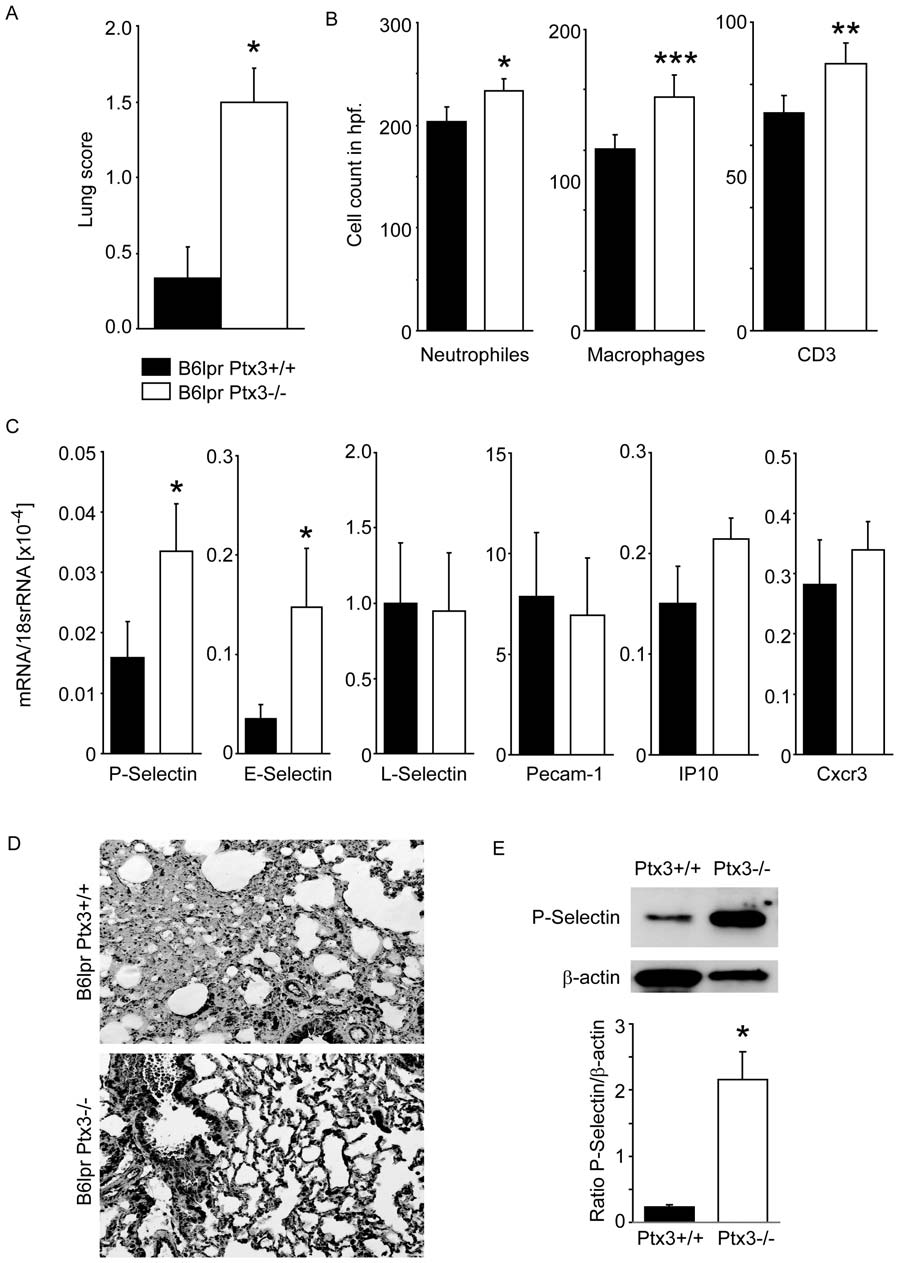
Lung damage and gene expression in B6*^lpr^* mice. A: Peribronchial and interstitial inflammation was scored from 0–4 as described in methods. Data represent means ± SEM of 8–12 mice in each group. B: Neutrophils, macrophages or T lymphocytes numbers were quantify, data represent means ± SEM from at least 12 mice in each group, * p<0.05, **p<0.005, ***p<0.0005 versus B6*^lpr^* mice. C: RNA was isolated from lungs of 6 months old B6*^lpr/Ptx3−/−^* and B6*^lpr^* wild-type mice for real-time RT-PCR. Data are expressed as means of the ratio of the specific mRNA from at least 5 mice versus that of 18S rRNA ± SEM. * p<0.05 versus B6*^lpr^* mice. D: Lung sections were stained with anti- P-selectin P. Images are representative for at least 12 mice in each group. Original magnification ×100. E: Protein samples were prepared from lungs of B6*^lpr^* or B6*^Ptx3−/−lpr^* mice. PTX3 Western blot indicates the quantitiative (20 µg protein load per lane) P-selectin protein expression. The histogram represents the ratio of P-selectin to the respective β-actin expression. Data represent means ± SEM from three independent experiments.

**Figure 9:**
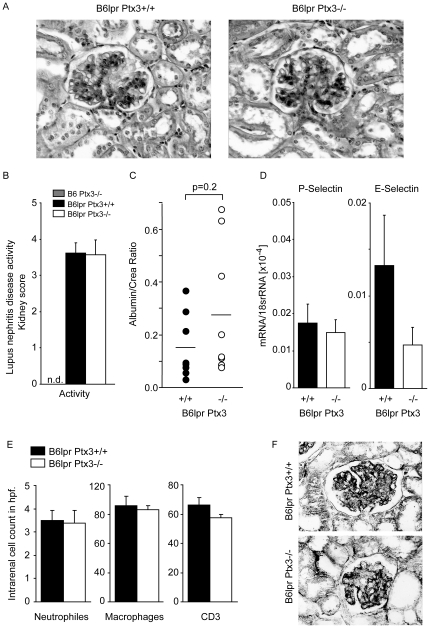
PTX3 and lupus nephritis in B6*^lpr^* mice. A: Kidney sections were stained with PAS. Images are representative for at least 12 mice in each group, original magnification ×400. B: The composite activity index of lupus nephritis (0–24) was assessed as described in methods. Data represent means ± SEM of 8–12 mice in each group. C: Urinary albumin/creatinine ratios were determined at 6 months as a functional marker of glomerular damage. D: Renal P-selectin and E-selectin mRNA levels were determined by real-time RT-PCR and are expressed as means of the ratio of the specific mRNA versus that of 18S rRNA ± SEM (n = 12 in each group). E: Neutrophils, macrophages or T lymphocytes numbers were quantify, data represent means ± SEM from at least 12 mice in each group. F: C9 complement staining was performed on kidney sections. No significant differences were detected between the two genotypes.

SLE may be associated with little or severe autoimmune tissue injury [32]. B6*^lpr^* mice do not develop major autoimmune tissue injuries although mild glomerulonephritis develops from 6 months of age [28]. *Ptx3*-deficient B6*^lpr^* mice revealed significant peribronchial and perivascular neutrophils, CD3+ T cell infiltrates accompanied by Mac2 + macrophages while significant pulmonary pathology was absent in age-matched B6*^lpr^* mice (Figure 7, 8A and 8B). Given the known role of PTX3 for P-selectin-mediated lung leukocyte recruitment we next determined the mRNA expression levels of P-, E-, and L-selectin, PECAM, IP10 and Cxcr3 in lungs of both mouse strains. Lack of PTX3 was associated with increased mRNA expression of P-selectin and E-selectin (but not L-selectin, PECAM, IP10 or Cxcr3) in lungs of 6 months old B6*^lpr^* mice (Figure 8C). Consistent with mRNA data, lack of PTX3 was associated with increased P-selectin expression on the protein level (Figures 8D and 8E). In contrast, the *Ptx3* Genotype had no effect on the activity of lupus nephritis, proteinuria, renal P-selectin and E-selectin mRNA expression, complement immunostaining, and renal leukocyte numbers (Figure 9). Together, PTX3 protects B6*^lpr^* mice from autoimmune lung disease but not from lupus nephritis

## Discussion

The long pentraxin PTX3, like the short pentraxins, has multiple regulatory roles on innate immunity. It modulates opsonization (including dead cell clearance), complement activation, and leukocyte recruitment, all processes that affect autoimmunity and autoimmune tissue injury [8]. The experimental strategy of generating *Ptx3*-deficient autoimmune B6*^lpr^* mice intended to test the role of *Ptx3* as a potential modifier gene for established autoimmunity. Our data now demonstrate that the evolution of autoimmunity and lupus autoantibodies in B6*^lpr^* mice seems to be independent of PTX3 but that PTX3 has a non-redundant role in surpressing autoimmune lung injury.

Interstitial or alveolar lung disease is rare in humans and usually absent in 6 months old B6*^lpr^* mice [33] but is well described in MRL*^lpr^* mice which suffer from more advanced lupus-like autoimmune tissue injuries at this age [34]. The peribronchial and perivascular lymphocyte infiltrates that we observed in B6*^lpr/Ptx3−/−^* mice were similar to those reported from MRL*^lpr^* mice and to those that we had previously observed in B6*^lpr^* mice with accelerated SLE [35]. Hence, lack of PTX3 specifically accelerated the evolution of autoimmune lung disease, albeit not kidney disease, in B6*^lpr^* mice. The knowledge about organ-specific pathomechanisms in SLE is limited. Kidney disease in autoimmune mice (and lupus patients) mainly develops from immune complex disease and depends on glomerular complement activation and macrophage recruitment [36]. By contrast, lung disease in MRL*^lpr^* mice involves the recruitment of CXCR3 positive T cells via local secretion of CXCL10 [37], and the endothelial expression of selectins [38] and intercellular adhesion molecule (ICAM)-1 [39]. In addition, TNF-α is a crucial mediator of lung injury in experimental lupus [40], [41]. Unspecific immunosuppressants like cyclophosphamide or dihydroorotate dehydrogenase inhibitors suppress pulmonary and renal manifestations in experimental lupus [42], [43]. However, the aforementioned molecular and cellular pathomechanisms of autoimmune lung injury differ, at least in part, from those of lupus nephritis because *Icam-1*-deficiency as well as TNF-α antagonism protects MRL*^lpr^* mice from lung but not from kidney disease [39], [41]. Furthermore, P-selectin is only induced in lungs but not in kidneys upon immune stimulation with LPS [38] or, as we found here, in experimental SLE. The latter finding is of particular interest because PTX3 was recently identified as an endogenous P-selectin inhibitor that limits leukocyte recruitment to the lung [27]. We therefore assume that the phenotype of *Ptx3*-deficient B6*^lpr^* mice underscores the organ-specific immunopathology in SLE and might relate to the potential of PTX3 to specifically impair leukocyte recruitment to the lung, e.g. by interacting with P-selectin.

Surprisingly, *Ptx3*-deficiency only marginally affected systemic autoimmunity in B6*^lpr^* mice. The *lpr* mutation induces autoimmunity by not allowing immature and potentially autoreactive T lymphocytes to undergo Fas-mediated apoptosis [28]. As a consequence CD4/CD8 double negative T cells accumulate and eventually undergo secondary necrosis which increases the exposure of potential lupus autoantigens to phagocytes and antigen-presenting cells [28], [44]. Our data clearly demonstrate that lack of PTX3 is associated with an additional expansion of CD4/CD8 double negative cells indicating that PTX3 has a non-redundant role in suppressing the expansion of this cell population in B6*^lpr^* mice. How PTX3 suppresses the expansion of the CD4/CD8 double negative T cells is less clear. Three studies consistently reported that soluble PTX3 impairs the capacity of cultured macrophages and dendritic cells to take up apoptotic cells which was concluded to counterbalance the opsonizing effect on dead cells of the short pentraxins [23], [25], [26]. A more recent study reported the opposite effect for membrane-bound PTX3 on apoptotic neutrophils which acts as an ‘eat me’ signal and fosters phagocytic clearance by macrophages [24]. The results from our own in-vivo phagocytosis assay are consistent with the latter report and document that PTX3 fosters the rapid clearance of apoptotic T cells by peritoneal macrophages, a process that may keep lupus autoantigens away from dendritic cells and avoid the activation of autoreactive T cells. Vice versa, impaired apoptotic cell clearance by antigen-presenting cells may serve as a stimulus for the selective expansion of CD4/CD8 double negative ‘autoreactive’ T cells in our study. It is of note that the numbers of CD4/CD8 double negative cells or their ratio to CD4/CD25/Foxp3 regulatory T cells are usually taken as a surrogate marker of the autoimmune process in experimental lupus [45], [46]. However, in our study the isolated expansion of the CD4/CD8 double negative T cells population was not associated with more lupus autoantibody production which raises doubts about their causal role for systemic autoimmunity.

Together, the long pentraxin PTX3 is required to suppress lung disease in systemic autoimmunity. PTX3 does not regulate lupus nephritis of B6*^lpr^* mice. Furthermore, PTX3 is redundant for the production of lupus autoantibodies in these mice, albeit it fosters the clearance of apoptotic cells and (thereby) inhibits the expansion of CD4/CD8 double negative T cells. These results add on to previous data that have documented organ-specific pathomechanisms for lupus manifestations. In addition, it is now intriguing to speculate that loss-of-function mutations in the *Ptx3* gene might represent a genetic risk factor for pulmonary manifestations of human SLE or that recombinant PTX3 or other PTX3 agonists might have the potential to specifically suppress autoimmune lung disease.

## Materials and Methods

### Animal studies

*Ptx3*-deficient mice were generated as previously described [47] and backcrossed to the C57BL/6 strain (B6, CharlesRiver Laboratories, Calco, Italy) to the N11 generation. B6*^Ptx3−/−^* and B6*^lpr^* mice (Charles River) were mated to generate B6*^lpr/Ptx3−/+^* mice which were then mated among each other to generate B6*^lpr/Ptx3+/+^* and B6*^lpr/Ptx3−/−^* mice as described [35]. Littermates female were used for all experimental procedures. In each individual mouse the genotype was assured by PCR. Mice were housed in groups of 5 mice in sterile filter top cages with a 12 hour dark/light cycle and unlimited access to autoclaved food and water. One cohort of mice was sacrificed by cervical dislocation at 24 weeks of age. All experimental procedures were performed according to the German animal care and ethics legislation and had been approved by the local government authorities (Regierung von Oberbayern, Az 55.2-1-54-2531-11-10).

### Phagocytose assay and generation of apoptotic cells

EL4 cells (1×10^6^/ml) were incubated with 90 µM of the DNA topoisomerase I inhibitor Camptothecin (Calbiochem, San Diego, USA) for 4 h at 37°C to induce apoptosis. Apoptosis was confirmed by flow cytometry after staining with FITC Annexin V Apoptosis Detection Kit 1 (BD Biosciences Pharmigen, San Diego, CA). After 4 h the apoptotic cells (1×10^6^/ml) were stained with 125 ng/µl pHrodo (Invitrogen, Eugene, OR). Mice were injected intraperitoneally with 500 µl of 4% thioglycollate medium (BD, Franklin Lakes, USA). After 73 h of incubation the mice were injected intraperitoneally with 200 µl of the apoptotic pHrodo-stained apoptotic EL4 cells. 40 min later peritoneal fluid were centrifuges and resuspended in HBSS (PAN-Biotech GmbH, Aldenbach, Germany) +4 mM EDTA (Biochrom KG, Berlin, Germany). The cells were stained with anti-F4/80 IgG (BD Pharmingen, Heidelberg, Germany) for flow cytometry analysis.

### Flow cytometry

Anti-mouse CD3, CD4, CD8 and CD25 antibodies (BD Pharmingen, Heidelberg, Germany) were used to detect CD3+CD4−CD8− double negative T cells and CD4+CD25+ regulatory T cells populations in spleens. Anti-CD11c was used to identify dendritic cells and their activation was assed by co-staining for CD40 (BD Pharmingen, Heidelberg, Germany). Anti-mouse B220, CD21, CD23, IgD, IgM antibodies (BD Pharmingen, Heidelberg, Germany) were used to detect mature B cells (B220+IgD+IgM+), marginal zone B cells (B220+CD21^high^CD23^low^) and follicular B cells (B220+CD21^low^CD23^high^). Plasma cells were identified by using anti-mouse antibodies for Ig κ light chain and CD138 (BD Pharmingen, Heidelberg, Germany). Respective isotype antibodies were used to demonstrate specific staining of cell subpopulations [35]. Quantification of cell number was done using counting beads for FACS (Invitrogen).

### Evaluation of autoimmune tissue injury

Lungs, spleens and kidneys from all mice were fixed in 10% buffered formalin, processed, and embedded in paraffin. Two µm sections for periodic acid-Schiff (PAS) stains were prepared following routine protocols [48]. The severity of the renal lesions was graded using the activity and chronicity indices for human lupus nephritis as described [49]. Autoimmune lung injury was scored semiquantitatively (0–4) by assessing the extent of peribronchial, perivascular or interstitial lymphocyte infiltrates as described [34]. For lung immunostaining: anti-mCD3e (clone 500A2, 1∶50), Mac2 (macrophages, Cederlane, Ontario, Canada, 1∶5000), rat anti-mouse neutrophils (Serotec, Oxford, UK, 1∶50) were used. For IgM staining of the spleen sections, anti-mouse IgM-mu-chain specific antibodies (Vector, Burlingame, CA) were used. The slides were scanned using an Olympus BX 61 microscope and recorded via CellP software.

Serum IL-12 levels were determined by ELISA following the manufacturer's protocols (OptEiA, BD, Biosciences, Heidelberg, Germany).

### Ptx3 protein expression analysis

Western blot analysis were performed from kidney, lung and spleen protein extracts, which were incubated in two times loading buffer for 5 min at 95°C, resolved by 10% SDS-PAGE, and transferred to an Immobilon-P membrane (Millipore, Eschborn, Germany). After blocking with 1% Western Blocking Reagent (Roche, Germany), the filter was incubated with Rat anti mouse Ptx3 antibody (1∶1000, Alpha Diagnostic International, San Antonio USA) overnight in TBS; β-actin (1∶1000, Cell Signaling Technology, Beverly, MA) for 1 hour), anti-P-selectin monoclonal antibody (mAb) RB40 (anti-P-selectin were prepared from hybridoma cells). Immune complexes were visualized using a peroxidase-conjugated anti-rat IgG Ab (1∶10000, Cell Signaling Technology, Beverly, MA) for 1 h and processed for detection by ECL (Amersham Pharmacia Biotech Europe, Freiburg, Germany).

### Autoantibody analysis

Serum antibody levels were determined by ELISA as described [50], [51]. Anti-dsDNA antibodies: NUNC maxisorp ELISA plates were coated with poly-L-lysine (Trevigen, Gaithersburg, MD, USA) and mouse embryonic stem cell dsDNA. After incubation with mouse serum, dsDNA-specific IgG, IgG_1_, IgG_2a/c_, IgG_2b_, IgG_3_ and serum IgG levels were detected by ELISA (Bethyl Labs, Montgomery, TX, USA). *Critidiae luciliae* assay: 1∶50 diluted serum was applied to fixed *C. luciliae* slides (BioRad Laboratories, Redmond USA.). Binding to *C. luciliae* kinetoplast was detected with FITC-conjugated goat anti–mIgG (1∶1000, Invitrogen, Oregon USA). DAPI staining (Vector Laboratories, Burlingame CA) allowed colocalization with kinetoplast dsDNA. For quantitation of kinetoplast staining intensity a semiquantitative score from 0–3 was used. Anti-Sm: NUNC maxisorp ELISA plates were coated with Smith (Sm) antigen (Immunovision, Springdale, AR). A horseradish peroxidase-conjugated goat anti-mouse IgG (Rockland, Gilbertsville, PA) was used for detection. The same procedure was followed for anti-SmRNP and anti-nucleosome antibodies as for anti-Sm except the ELISA plates were captured with Sm-RNP complex (Immunovision) or dsDNA together with histones (USB Corporation, Ohio USA) respectively instead of Sm antigen. Rheumatoid factor: ELISA plates were coated with 10 µg/ml IgG (Jackson Immunoresearch, West Grove, PA, USA) overnight at 4°C. C57BL/6 10 week mouse serum was used as negative control. HRP conjugated anti-mouse IgG was used as secondary antibody.

### Real-time quantitative RT-PCR

Real time RT-PCR was performed on mRNA from mouse organs as previously described [52]. SYBR Green Dye detection system was used for quantitative real-time PCR on Light Cycler 480 (Roche, Mannheim, Germany). All the technical steps were performed according to The Minimum Information for Publication of Quantitative Real-Time PCR Experiments (MIQE) guidelines [53]. For relative quantification the 2^−ΔΔCt^ analysis method was used. Controls consisting of ddH2O were negative for target and housekeeper genes. Gene-specific primers (300 nM, Metabion, Martinsried, Germany) were designed to be cDNA specific and to target possibly all known transcripts of gene of interest. In silico specificity screen (BLAST) was performed. PTX3: ID NM_008987 right: 5′-CCTGCTTTGTGCTCTC TGGT-3′, left: 5′-TCTCCAGCATGATGAACAGC-3′; E-Selectin: ID NM_011345 right: 5′-TCTATTTCCCACGATGCATTT-3′, left: 5′-CTGCCAAAGCCTTCAATCAT-3′; L-Selectin: ID NM_011346 right: 5′-TTCATGGCTTTCCTTTCACA–3′; left: 5′-CTGGCATTTCTCATT TGGCT-3′; P-Selectin: ID NM_011347 right: 5′-GGACACTTGATGGCTTCACA-3′, left: 5′-CAGTTCATGTGCGATGAAGG-3′; PECAM-1: ID NM_001032378 right: 5′-TCCTTCCTGCTTCTTGCTAGCT-3′, left: 5′-GAGCCCAATCACGTTTCAGTTT-3′; IP-10: ID NM_021274 right: 5′-GGCTGGTCACCTTTCAGAAG-3′, left: 5′-ATGGATGGACAGCAGAGAGC-3′; Cxcr3: ID NM_009910, right: 5′- TCTCGTTTTCCCCATAATCG-3′, left: 5′-AGCCAAGCCATGTACCTTGA-3′.

### Statistical analysis

One-way ANOVA followed by post-hoc Bonferroni's test was used for multiple comparisons using GraphPad Prism 4.03 version. Single groups were compared by unpaired two-tailed Students t-test or non-parametric Mann-Whitney test. Data were expressed as mean ± SEM. Statistical significance was assumed at a p value of <0.05.
